# HSC-MET: Heterogeneous signcryption scheme supporting multi-ciphertext equality test for Internet of Drones

**DOI:** 10.1371/journal.pone.0274695

**Published:** 2022-09-29

**Authors:** Xiaodong Yang, Ningning Ren, Aijia Chen, Zhisong Wang, Caifen Wang

**Affiliations:** 1 Department of Computer Science and Engineering, Northwest Normal University, Lanzhou, Gansu, China; 2 Department of Big Data and Internet, Shenzhen Technology University, Shenzhen, Guangdong, China; Jazan University Faculty of Computer Science, SAUDI ARABIA

## Abstract

Internet of Drones (IoD) is considered as a network and management architecture, which can enable unmanned aerial vehicles (UAVs) to collect data in controlled areas and conduct access control for UAVs. However, the current cloud-assisted IoD scheme cannot efficiently achieve secure communication between heterogeneous cryptosystems, and does not support multi-ciphertext equality tests. To improve the security and performance of traditional schemes, we propose a heterogeneous signcryption scheme (HSC-MET) that supports multi-ciphertext equality test. In this paper, we use a multi-ciphertext equality test technique to achieve multi-user simultaneous retrieval of multiple ciphertexts safely and efficiently. In addition, we adopt heterogeneous signcryption technology to realize secure data communication from public key infrastructure (PKI) to certificateless cryptography (CLC). At the same time, the proposed scheme based on the computation without bilinear pairing, which greatly reduces the computational cost. According to the security and performance analysis, under the random oracle model (ROM), the confidentiality, unforgeability and number security of HSC-MET are proved based on the computational Diffie-Hellman (CDH) problem.

## Introduction

Unmanned aerial vehicles(UAVs) [1, 2] as devices using radio remote control technology and self-provided program control mechanism, have the advantages of small size, low cost, and flexible deployment. As a result, it is widely used in film and television shooting, environmental monitoring, and smart farms. To provide coordinated and orderly access for UAVs, the Internet of Drones(IoD) [3–5] came into being. IoD is a sophisticated heterogeneous network containing a large number of sensors and actuators. In IoD environment, entities communicate through open wireless channels, thus facing many privacy and security issues [6]. Entities in IoD also have limited computing and storage capabilities, so it is extremely important to design an efficient and secure algorithm. Bharany et al. [7] proposed a clustering protocol for flying ad-hoc networks (FANETs) based on a moth flame optimization algorithm for safe and efficient UAV access. It ensures UAVs’ efficient and safe access while also improving FANET fault tolerance. Bharany et al. [8] proposed a unique clustering algorithm EE-SS for FANETs to increase the service life of UAVs in forest fire detection, which reduced cluster head overhead and improved system efficiency. With the wide application of UAVs, the storage and processing of big data in IoD have become a top priority. Fortunately, cloud computing technology can provide users with computing services regardless of time and place. However, since cloud servers are not trusted, data is usually encrypted or signcrypted and stored in cloud servers, which makes efficient data retrieval difficult.

To ensure the security of UAVs, Bera et al. [9] proposed a blockchain-based secure access control scheme to achieve authentication between drones and between drones and a ground station server. The scheme satisfies the immutability of data. Hussain et al. [10] proposed an authentication scheme based on elliptic curve cryptography to secure the communication between a data user and a drone. Khan et al. [11] proposed an identity-based proxy signcryption scheme based on hyperelliptic curves. The scheme allows for outsourced decryption to reduce the computational cost. They proved that the scheme satisfies indistinguishability against adaptive selected scrambled text attacks and existential forgery for adaptive selected plaintext attacks under the ROM. Gope and Sikdar [12] proposed an efficient privacy-aware authenticated key agreement scheme for edge-assisted IoD. The scheme does not need to store any secret keys in the devices but still can provide the desired security features. But the IoD is a heterogeneous and complex network, so these schemes in [9–12] are inapplicable. To realize secure communication between heterogeneous cryptosystems, Sun and Li [13] proposed a heterogeneous signcryption scheme (HSC), which realized the secure communication from public key infrastructure (PKI) to identity-based cryptography (IBC). Inspired by Sun and Li, many HSC schemes have been proposed [14–20].

Although the schemes proposed in [14–20] have realized the secure communication between heterogeneous cryptosystems, it does not consider the efficient retrieval of ciphertexts. Cloud storage has brought great convenience, but this approach reduces the availability of data. Boneh et al. [21] proposed to use keyword search-based public key encryption (PKE-KS) to realize ciphertext retrieval in cloud servers, but it only supports retrieval of ciphertext encrypted with the same public key. To improve this limitation, Yang et al. [22] proposed a public key encryption scheme that supports the ciphertext equality test (PKE-ET), which allows users to compare two ciphertexts obtained by using the different public keys. Subsequently, scholars have proposed a series of similar schemes [23–27], but these schemes only support the equality test after dividing two ciphertexts into a group. Therefore, it faces the challenges of low retrieval efficiency and high computational cost. To reduce computational cost and improve the efficiency of ciphertext retrieval, Susilo et al. [28] proposed public-key encryption with flexible multi-ciphertext equality test (PKE-FMET). Although this scheme supports the equality test of more than two ciphertexts, there are problems such as not satisfying message authentication and communication between heterogeneous cryptosystems.

### Our contributions

With the motivation of solving the above-mentioned problems, we present a heterogeneous signcryption scheme that supports the multi-ciphertext equality test (HSC-MET). The main contributions are as follows.

1. Our scheme utilizes heterogeneous signcryption technology to realize secure communication from PKI to certificateless public key cryptography (CLC), eliminating the limitation of existing schemes that only support communication in the same cryptosystem.
2. We adopt the multi-ciphertext equality testing technique to address the limitations of pairwise ciphertext equality testing to reduce the computational cost required for ciphertext equality testing in multi-user and multi-ciphertext environments.
3. Our scheme is based on computation without bilinear pairing, which greatly reduces the computing cost and improves the communication and retrieval efficiency for the problem of limited computing resources of UAVs.
4. Our scheme is proven to meet unforgeability and confidentiality based on the CDH problem under the ROM. We demonstrated our scheme’s number security using the definition of a new security number-security proposed in [26].
5. We compared our scheme with similar schemes in terms of confidentiality, unforgeability, and computational costs. Analysis results show that our scheme meets higher confidentiality, unforgeability and lower computational costs.

### Organization

The rest of this paper is structured as follows. The complexity assumption, Kramer’s rule, Vandermonde determinant, formal definition, and security design are all introduced in section 2. The system design is presented in section 3. In section 4, we go over the algorithm processes of the HSC-MET scheme in detail. section 5 describes our scheme’s correct analyses. Our scheme’s securities were proven in section 6. Section 7 then compares the performance of our scheme to existing similar schemes in terms of efficiency and function. Finally, in section 8, we summarize the paper’s conclusion.

### Related work

Table 1 summarizes the functional properties, confidentiality, and unforgeability analyses of the references [13–28].

**Table 1:** Characteristics of various works.

Schemes	ET	MET	SC	HSC	Without bilinear pairing	Cryptosystem	Confidentiality	Unforgeability
Sun et al. [13]	×	×	✓	✓	×	PKI→IBC	IND-CCA	EUF-CMA
Elkhalil et al. [14]	×	×	✓	✓	✓	CLC→PKI	IND-CCA2	EUF-CMA
Ali et al. [15]	×	×	✓	✓	×	IBC→PKI	IND-CCA2	EUF-CMA
Qiu et al. [16]	×	×	✓	✓	✓	IBC→CLC	IND-CCA2	EUF-CMA
Cao et al. [17]	×	×	✓	✓	×	PKI↔IBC	IND-CCA2	EUF-CMA
Wang et al. [18]	×	×	✓	✓	×	PKI↔IBC	IND-CCA2	EUF-CMA
Luo et al. [19]	×	×	✓	✓	✓	PKI↔CLC	IND-CCA2	EUF-CMA
Ji et al. [20]	×	×	✓	✓	×	PKI↔IBC	IND-CCA2	EUF-CMA
Boneh et al. [21]	×	×	×	×	×	PKE-KS	IND-CCA	×
Yang et al. [22]	✓	×	×	×	×	Public key encryption	IND-CCA	×
Rashad et al. [23]	✓	×	×	×	×	CLC encryption	IND-CCA	×
Li et al. [24]	✓	×	×	×	×	Proxy re-encryption	IND-CCA	×
Xiong et al. [25]	✓	×	✓	✓	×	PKI→IBC	IND-CCA2	EUF-CMA
Xiong et al. [26]	✓	×	✓	✓	×	IBC→PKI	IND-CCA2	EUF-CMA
Hou et al. [27]	✓	×	✓	✓	×	PKI→CLC	IND-CCA	EUF-CMA
Susilo et al. [28]	✓	✓	×	×	✓	Public key encryption	IND-CPA	×

×: not supported;

✓: supported.

IND-CCA: Indistinguishability against Chosen Ciphertext Attack.

IND-CCA2: Indistinguishability against Adaptive Chosen Ciphertext Attack.

IND-CPA: Indistinguishability against Chosen Plaintext Attack.

EUF-CMA: Existential Unforgearility against Chosen Message Attack.

The concept of heterogeneous signcryption (HSC) was proposed by Sun and Li [13]. Although their scheme realizes the heterogeneous communication from PKI to IBC, it has low security performances and high computational costs. Inspired by Sun and Li, many scholars have studied HSC. Eltayeeb et al. [14] proposed a HSC scheme without pairing computation, which realizes secure communication from CLC to PKI. Ali et al. [15] designed a HSC scheme from IBC to PKI to realize heterogeneous communication between vehicles and other entities in VANETs. The scheme supported the receivers to decrypt messages in batches, which greatly reduced the computational cost. Qiu et al. [16] proposed a HSC scheme based on the dense communication and heterogeneity of the intelligent mobile Internet of Things, which realized secure communication from IBC to CLC. The proposed scheme does not need to perform bilinear pairing operations and outsources part of the verification operations to the gateway, which greatly reduces the calculation and communication overhead of the sender and the receiver. Cao et al. [17] proposed an improved mutual HSC scheme between PKI and IBC for problems, in which the scheme of Wang et al. [18] could not resist attacks. They analyzed the security of the proposed scheme based on the assumption of the CDH problem. However, the scheme of Cao et al. uses bilinear pairing, which has a significant computational overhead. Luo et al. [19] proposed a mutual HSC scheme based on different system parameters for 5G network slices, which realized the mutual communication between CLC and PKI cryptosystem and satisfied the anonymity of messages. Ji et al. [20] proposed a mutual HSC scheme based on PKI and IBC, and proved the confidentiality and unforgeability of the scheme based on the q-Diffie-Hellman inverse problem.

The concept of PKE-ET was proposed by Yang et al. [22]. A tester can determine whether the underlying plaintext corresponding to two ciphertexts encrypted with different public keys is equal according to [22]. It has attracted the attention of many scholars. Rashad et al. [23] proposed CL-PKC-ET, a certificateless public key cryptography with equality test, to support the ciphertext equality test in IoV. Li et al. [24] designed a cryptographic scheme in IoT-based healthcare systems using proxy re-encryption and ciphertext equality test technology. The scheme realizes the flexible sharing of medical data. Shen et al. [29] proposed a group public key encryption scheme supporting equality test without bilinear pairings, G-PKEET, which greatly reduces the computational overhead. In [22–24, 29], anyone can perform the equality test algorithm on two ciphertexts, which brings many security risks. Therefore, there are many authorized equality test schemes were presented [30–32], in which only the authorized tester is promised to execute the equality test algorithm. Furthermore, some equality test schemes for heterogeneous systems have been proposed. Xiong et al. [25] proposed a HSC scheme supporting the ciphertext equality test for Internet of Things (IIOT). The scheme realizes a flexible ciphertext equality test under heterogeneous communication between sensors in PKI and cloud server in IBC. They also prove the security of the scheme in ROM. Xiong et al. [26] proposed a HSC scheme from IBC to PKI with equality test (HSCIP-ET), which realized secure communication between sensors and data users. According to the IoT application scenario, Hou et al. [27] proposed a HSC scheme supporting the ciphertext equality test, which realized secure communication between PKI and CLC. In [25–27], the schemes only support equality testing after grouping two ciphertexts and have many bilinear pairing operations. As a result, they face the challenges of low retrieval efficiency and high computational costs. Susilo et al. [28] proposed public-key encryption with flexible multi-ciphertext equality test (PKE-FMET) to achieve efficient ciphertext retrieval in multi-user scenarios.

## Preliminaries

### Complexity assumption

**Definition 1.** Computational Diffie-Hellman (CDH) problem [27]: Given a group *G*, and (*P*, *aP*, *bP*) ∈ *G*, computing *abP* ∈ *G*, where $a,b\in Z_{q}^{*}$.

### Cramer’s rule

For the non-homogeneous linear equation set $\left\{ \begin{array}{c} a_{1,1}x_{1}+a_{1,2}x_{2}+\cdots +a_{1,n}x_{n}=b_{1}\\ a_{2,1}x_{1}+a_{2,2}x_{2}+\cdots +a_{2,n}x_{n}=b_{2}\\ \vdots \\ a_{n,1}x_{1}+a_{n,2}x_{2}+\cdots +a_{n,n}x_{n}=b_{n} \end{array},\right.$ its coefficient determinant is $\text{det}\left(V\right)=|\begin{array}{cccc} a_{1,1} & a_{1,2} & \cdots & a_{1,n}\\ a_{2,1} & a_{2,2} & \cdots & a_{2,n}\\ \vdots & \vdots & \ddots & \vdots \\ a_{n,1} & a_{n,2} & \cdots & a_{n,n} \end{array}|.$ If det(*V*) ≠ 0, then there is a unique solution for the equation set.

### Vandermonde determinant

The matrix of the form $V=[\begin{array}{ccccc} 1 & a_{1} & a_{1}^{2} & \cdots & a_{1}^{n-1}\\ 1 & a_{2} & a_{2}^{2} & \cdots & a_{2}^{n-1}\\ \vdots & \vdots & \vdots & \ddots & \vdots \\ 1 & a_{n} & a_{n}^{2} & \cdots & a_{n}^{n-1} \end{array}]$ called the Vandermonde matrix, and the corresponding Vandermonde determinant is $\text{det}\left(V\right)=|\begin{array}{ccccc} 1 & a_{1} & a_{1}^{2} & \cdots & a_{1}^{n-1}\\ 1 & a_{2} & a_{2}^{2} & \cdots & a_{2}^{n-1}\\ \vdots & \vdots & \vdots & \ddots & \vdots \\ 1 & a_{n} & a_{n}^{2} & \cdots & a_{n}^{n-1} \end{array}|=\prod_{1\leq i<j\leq n}\left(a_{i}-a_{j}\right)$.

### Formal definition

The HSC-MET scheme consists of the following algorithms.

1. **Setup:** Input the system security parameter λ, and the key generation center (KGC) and certificate authority (CA) output the system master key *s* and system parameter *para*. The KGC publicizes *para* and keeps *s* secretly.
2. **PKI-Gen:** Input the identity *ID*_*p*_ of the PKI system user, and the CA outputs a digital certificate.
3. **CLC-PGen:** Input the system parameter *para* and identity *ID*_*c*_ of the CLC system user, and the KGC outputs the partial private-public key pair.
4. **CLC-SSV:** *ID*_*c*_ selects $s_{2}\in Z_{q}^{*}$ randomly and sets it as a secret value.
5. **CLC-CGen:** Input the system parameter *para*, the secret value *s*_2_, partial private-public key pair (*SK*_*c*1_, *PK*_*c*1_), and the user outputs the complete private-public key pair (*SK*_*c*_, *PK*_*c*_).
6. **Trapdoor:** Input the private key *SK*_*c*_, and the user outputs *td*_*c*_ as trapdoor.
7. **Signcryption:** Input the system parameter *para*, the plaintext message *m*, the receiver’s public key *PK*_*c*_, and the sender’s private key *SK*_*p*_, and the sender calculates the ciphertext *δ*.
8. **Unsigncryption:** Input the system parameter *para*, ciphertext *δ*, receiver’s private key key *SK*_*c*_ and sender’s public key *PK*_*p*_, and the receiver outputs the plaintext message *m* or error symbol ⊥.
9. **Test:** Input ciphertexts *δ*_*i*_ and trapdoors *td*_*i*_ where *i* ∈ {1, 2, ⋯, *t*}, the cloud server outputs error symbol ⊥ or multi-ciphertext equality test result 0/1.

### Security model

In the ROM, the HSC-MET scheme needs to meet the confidentiality of the message, IND-CCA2, and the unforgeability of ciphertext, EUF-CMA.

#### Confidentiality

We define two types of adversaries, Type-1 and Type-2. A Type-1 adversary $A_{1}$ does not know the system master key, but can replace any user’s public key. A Type-2 adversary $A_{2}$ can obtain the system master key, but cannot replace any user’s public key.

**Definition 2.** If no Type-1 adversary $A_{1}$ wins game 1 with a non-negligible advantage in PPT, the HSC-MET scheme satisfies IND-CCA2–1.

**Game 1.** The game process between challenger C and adversary $A_{1}$ is as follows.

**Setup:** C executes the setup algorithm, outputs the system parameter *para* and the master key *s*, returns *para* to $A_{1}$, and stores *s* secretly.

**Phase 1:** $A_{1}$ can perform limited following polynomial queries.

- **Partial private key query:** $A_{1}$ queries for the partial private key of *ID*_*c*_. C executes the CLC-PGen algorithm to generate *SK*_*c*1_ and return it to $A_{1}$.
- **Private key query:** $A_{1}$ queries for the private key of *ID*_*c*_. C executes the CLC-CGen algorithm to generate (*SK*_*c*_, *PK*_*c*_) and return *SK*_*c*_ to $A_{1}$.
- **Public key query:** $A_{1}$ queries for the public key of *ID*_*c*_. C executes the CLC-CGen algorithm to generate (*SK*_*c*_, *PK*_*c*_) and return *PK*_*c*_ to $A_{1}$.
- **Replace public key query:** $A_{1}$ can select any public key $P{K_{c2}}^{*}$ to replace the original public key *PK*_*c*2_.
- **Trapdoor query:** $A_{1}$ queries for the trapdoor of *ID*_*c*_. C executes the Trapdoor algorithm to generate *td*_*c*_ and return it to $A_{1}$.
- **Signcryption query:** When receiving the query with (*m*_*i*_, *ID*_*pi*_, *ID*_*ci*_) submitted by $A_{1}$, C executes the Signcryption algorithm to generate *δ*_*i*_, and returns it to $A_{1}$.
- **Unsigncryption query:** When receiving the query with (*ID*_*pi*_, *ID*_*ci*_, *δ*_*i*_) submitted by $A_{1}$, C executes the Unsigncryption algorithm to generate *m*_*i*_, and returns it to $A_{1}$.

**Challenge:** $A_{1}$ selects the sender’s identity $I{D_{p}}^{*}$, receiver’s identity $I{D_{c}}^{*}$ and two plaintexts of equal length *m*_0_ and *m*_1_ to C. C selects randomly *ξ* ∈ {0, 1} and performs the signcryption algorithm to generate ciphertext δ* and return it to $A_{1}$.

**Phase 2:** After receiving δ*, the adversary $A_{1}$ continues to execute the queries in Phase 1. However, $A_{1}$ can neither query the private key of $I{D_{c}}^{*}$, nor can $A_{1}$ make unsigncryption query of $\left(\delta^{*},I{D_{p}}^{*},I{D_{c}}^{*}\right)$. $A_{1}$ also can’t query the trapdoor of $I{D_{c}}^{*}$.

**Guess:** $A_{1}$ outputs a guess value ξ* ∈ {0, 1}. $A_{1}$ wins the game if ξ* = *ξ*. We define the advantage of $A_{1}$ as $Adv_{A_{1}}^{\text{IND}-\text{CCA}2-1}\left(\lambda \right)=\left|\text{Pr}\left[\xi^{*}=\xi \right]-\frac{1}{2}\right|$, where Pr[ξ* = *ξ*] represents the probability of ξ* = *ξ*.

**Definition 3.** If no Type-2 adversary $A_{2}$ wins game 2 with a non-negligible advantage in PPT, the HSC-MET scheme satisfies IND-CCA2–2 security.

**Game 2.** The game process between challenger C and adversary $A_{2}$ is as follows.

**Setup:** C executes the setup algorithm, outputs the system parameter *para* and the master key *s*, and returns them to $A_{2}$.

**Phase 1:** $A_{2}$ can perform all the queries in Definition 2 except the replace public key query.

The challenge, phase 2, and guess stage are the same as Definition 2 and will not be repeated here. We define the advantage of $A_{2}$ as $Adv_{A_{2}}^{\text{IND}-\text{CCA}2-2}\left(\lambda \right)=\left|\text{Pr}\left[\xi^{*}=\xi \right]-\frac{1}{2}\right|$ where Pr[ξ* = *ξ*] represents the probability of ξ* = *ξ*.

#### Unforgeability

**Definition 4.** If no adversary F wins Game 3 with a non-negligible advantage *ε* in PPT, it is said that the HSC-MET scheme can satisfy EUF-CMA security.

**Game 3.** The game between challenger C and adversary F is as follows.

**Training:** F can perform limited following polynomial queries.

- **Key query:** F queries for the public key of *ID*_*p*_, and C executes the PKI-Gen algorithm to generate (*SK*_*p*_, *PK*_*p*_) and return to F.
- **Signcryption query:** When receiving the query with (*m*_*i*_, *ID*_*pi*_, *ID*_*ci*_) submitted by F, C executes the signcryption algorithm to generate *δ*_*i*_, and returns it to F.
- **Unsigncryption query:** When receiving the query with (*ID*_*pi*_, *ID*_*ci*_, *δ*_*i*_) submitted by F, C executes the unsigncryption algorithm to obtain *m*_*i*_, and returns it to F.

**Forgery:** F selects the sender’s identity $I{D_{p}}^{*}$ and the receiver’s identity $I{D_{c}}^{*}$, and forges a ciphertext δ*. If δ* can meet the following requirements, F can win the Game 3.

The error symbol ⊥ will not be returned when the unsigncryption query is performed on $\left(\delta^{*},I{D_{p}}^{*},I{D_{c}}^{*}\right)$.
The adversary F can not query for the private key $S{K_{p}}^{*}$ of the $I{D_{p}}^{*}$.
δ* cannot be generated by the signcryption query of $\left(m^{*},I{D_{p}}^{*},I{D_{c}}^{*}\right)$.

We define the advantage of F to win in this game as $Adv_{F}^{\text{EUF}-\text{CMA}}\left(\lambda \right)=Pro\left[Fwins\right]$.

## Scheme design

**Fig 1:**
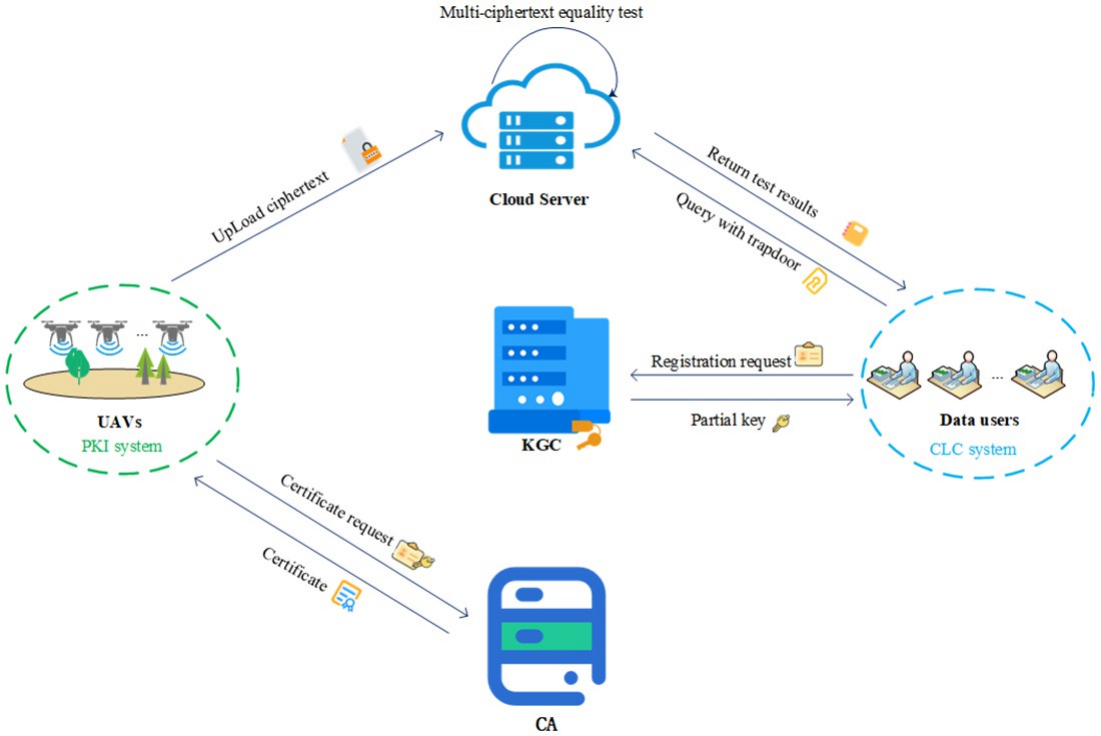
System model.

1. **Research questions and methodologies:** Table 2 displays the main research problems and relevant solutions of this paper, which are based on the previous relevant work subsection’s collections and analyses of references.
2. **Scheme processes:**
  - **Setup:** The KGC and CA initialize the system and generate the system parameters.
  - **User-Gen:** The CA generates digital certificates for UAVs in PKI. The KGC generates partial keys for data users in CLC.
  - **Signcrypt and upload:** UAVs signcrypt the collected data and upload it to the cloud server.
  - **Test:** The cloud server performs the equality test for multi-ciphertexts.
  - **Download and unsigncrypt:** Data users download and unsigncrypt data from the cloud server.
3. **System model:** The system model of our scheme is composed of five entities: KGC, CA, UAVs, cloud server and data users. The functions of each entity are as follows. The system model diagram is shown in Fig 1.
  - **KGC.** The KGC initializes the system, generates the key and system parameter, and distributes partial keys to data users.
  - **CA.** The CA issues digital certificates for UAVs.
  - **UAVs.** UAVs collect and signcrypt the collected environmental data, and upload it to the cloud server.
  - **Cloud server.** The cloud server stores the uploaded ciphertext, and processes the data user’s request to execute the test algorithm, and returns the test result to the users.
  - **Data users.** Users who wish to obtain environmental data, such as monitoring personnel and data processing centers, are responsible for submitting the trapdoor of the ciphertext equality test to the cloud server and verifying the ciphertext that meets the requirements.

**Table 2:** Research questions and solutions.

No.	Problem	Solution
1	How can data confidentiality and unforgeability be achieved at the same time?	Signcryption
2	How to realize the secure communication between heterogeneous entities in IoD?	Heterogeneous signcryption
3	How to achieve secure storage of data on cloud servers?	Store ciphertexts
4	How to efficiently retrieve the ciphertexts on the cloud server?	Multi-ciphertext equality test
5	How to reduce the computational cost of the scheme?	Without bilinear pairing

## Our construction

1. **Setup:** Given the system security parameter λ. KGC selects a large prime number *q*(*q* ≥ 2^λ^) and an additive cyclic group *G* with order *q* and generator *P*. Four hash functions, $H_{1}:{\left\{0,1\right\}}^{*}\to {Z_{q}}^{*}$, *H*_2_: *G* → {0, 1}^2*l*^, *H*_3_: *G* → {0, 1}^*nl*^ and *H*_4_: {0, 1}* → {0, 1}^λ^ are defined. KGC randomly selects $s\in Z_{q}^{*}$ as the system master key *SK* and calculates the public key *PK* = *sP*. It also selects the maximum number of ciphertexts that can perform the multi-ciphertext equality test, *n*. KGC sets and exposes *para* = {λ, *G*, *q*, *P*, *PK*, *H*_1_, *H*_2_, *H*_3_, *H*_4_, *n*}.
2. **PKI-Gen:** *ID*_*p*_ selects $s_{p}\in Z_{q}^{*}$ randomly, and calculates *PK*_*p*_ = *s*_*p*_*P*. The user sends (*ID*_*p*_, *PK*_*p*_) to CA which generates a digital certificate for it.
3. **CLC-PGen:** When receiving the registration request from *ID*_*c*_, KGC randomly selects $s_{1}\in Z_{q}^{*}$, and calculates *PK*_*c*1_ = *s*_1_*P* and *SK*_*c*1_ = *s*_1_ + *SKH*_1_(*ID*_*c*_). Then the KGC returns (*SK*_*c*1_, *PK*_*c*1_) to *ID*_*c*_ securely.
4. **CLC-SSV:** *ID*_*c*_ randomly selects $s_{2}\in Z_{q}^{*}$ as a secret value.
5. **CLC-CGen:** *ID*_*c*_ sets *SK*_*c*2_ = *s*_2_, *PK*_*c*2_ = *s*_2_*P*, *SK*_*c*_ = (*SK*_*c*1_, *SK*_*c*2_) and *PK*_*c*_ = (*PK*_*c*1_, *PK*_*c*2_).
6. **Trapdoor:** Input the private key *SK*_*c*_ = (*SK*_*c*1_, *SK*_*c*2_), output the *td*_*c*_ = *SK*_*c*2_.
7. **Signcryption:** Input (*para*, *m*, *PK*_*c*_, *SK*_*p*_) and output *δ*. Specific steps are as follows.
  - Calculate *f*_0,*n*_ = *H*_1_(*m*||*n*) and *f*_*i*,*n*_ = *H*_1_(*m*||*n*||*f*_0,*n*_||⋯||*f*_*i* − 1, *n*_) where *i* ∈ {1, 2, ⋯, *n* − 1}.
  - Calculate *f*_*i*,*j*_ = *H*_1_(*f*_*i*,*j*+1_) where *i* ∈ {*k*, ⋯, *n* − 1} & j ∈ {0, 1, ⋯, *i* − 1}. And calculate *f*_*i*_(*x*) = *f*_0,*i*_ + *f*_1,*i*_ *x* + ⋯ + *f*_*i* − 1, *i*_ *x*^*i*−1^, *i* ∈ {*k*, ⋯, *n*}, where *k* is the number of ciphertexts that can be tested for equality.
  - Select $r,X\in Z_{q}^{*}$ randomly. Calculate *Y* = *SK*_*p*_(*PKH*_1_(*ID*) + *PK*_*c*1_) and *R* = *rPK*_*c*2_.
  - Calculate *C*_1_ = *rP*, *C*_2_ = (*m*||*r*) ⊕ *H*_2_(*Y*) ⊕ *H*_2_(*R*), *C*_3_ = (*X*||*f*_*k*_(*X*)||⋯ ||*f*_*n*_(*X*)) ⊕ *H*_3_(*R*) and *C*_4_ = *H*_4_(*C*_1_||*C*_2_||*C*_3_||*f*_0,*k*_||*f*_1,*k*_||⋯||*f*_*k* − 1, *k*_||*R*||*k*).
  - Output the ciphertext *δ* = (*C*_1_, *C*_2_, *C*_3_, *C*_4_, *k*).
8. **Unsigncryption:** Input (*para*, *δ*, *PK*_*p*_, *SK*_*c*_) and output *m*′ or ⊥. Specific steps are as follows.
  - Calculate $Y^{{}^{\prime }}=SK_{c1}PK_{p}$, $R^{{}^{\prime }}=SK_{c2}C_{1}$ and $m^{{}^{\prime }}\left|\right|r^{{}^{\prime }}=H_{2}\left(Y^{{}^{\prime }}\right)⊕H_{2}\left(R^{{}^{\prime }}\right)$ ⊕ *C*_2_.
  - Calculate ${f^{{}^{\prime }}}_{0,n}=H_{1}(m^{{}^{\prime }}\left||n\right)$ and ${f^{{}^{\prime }}}_{i,n}=H_{1}(m^{{}^{\prime }}\left||n|\right|f_{0,n}^{{}^{\prime }}\left||\cdots |\right|f_{i-1,n}^{{}^{\prime }})$ where *i* ∈ {1, 2, ⋯, *n* − 1}.
  - Calculate ${f^{{}^{\prime }}}_{i,j}=H_{1}\left({f^{{}^{\prime }}}_{i,j+1}\right)$ where *i* ∈ {*k*, ⋯, *n* − 1} & j ∈ {0, 1, ⋯, *i* − 1} and $X^{{}^{\prime }}\left|\right|{f^{{}^{\prime }}}_{k}\left(X\right)||\cdots ||{f^{{}^{\prime }}}_{n}\left(X\right)=C_{3}⊕H_{3}\left(R^{{}^{\prime }}\right)$.
  - Verify that the Eqs (1), (2) and (3) are true, where *i* ∈ {*k*, ⋯, *n*}.
    $$\begin{array}{c} C_{1}=r^{{}^{\prime }}P \end{array}$$ (1)
    $$\begin{array}{c} {f^{{}^{\prime }}}_{i}\left(X^{{}^{\prime }}\right)={f^{{}^{\prime }}}_{0,i}+{f^{{}^{\prime }}}_{1,i}X^{{}^{\prime }}+\cdots +{f^{{}^{\prime }}}_{i-1,i}{X^{{}^{\prime }}}^{i-1} \end{array}$$ (2)
    $$\begin{array}{c} C_{4}=H_{4}(C_{1}\left|\right|C_{2}\left|\right|C_{3}\left|\right|{f^{{}^{\prime }}}_{0,k}\left|\right|{f^{{}^{\prime }}}_{1,k}\left||\cdots |\right|{f^{{}^{\prime }}}_{k-1,k}\left|\right|R^{{}^{\prime }}\left||k\right) \end{array}$$ (3)
    If the equations are all true, return $m^{{}^{\prime }}$. Otherwise, return ⊥.
9. **Test:** Input *t* ciphertexts *δ*_*i*_ = (*C*_*i*__,1_, *C*_*i*,__2_, *C*_*i*_, _3_, *C*_*i*,__4_, *k*_*i*_) and trapdoors *td*_*i*_. Let *k* = max{*k*_1_, *k*_2_, ⋯, *k*_*t*_}. If *k* ≤ min{*t*, *n*}, perform the following computations. Otherwise, return ⊥.
  - Calculate $X_{i}\left|\right|f_{i,k_{i}}\left(X_{i}\right)\left||\cdots |\right|f_{i,n}\left(X_{i}\right)=C_{i,3}⊕H_{3}\left(td_{i}C_{i,1}\right)$ and extract *f*_1,*k*_(*X*_1_), *f*_2,*k*_(*X*_2_), ⋯, *f*_*k* − 1, *k*_(*X*_*k*−1_) from the ciphertexts.
  - Assume that the plaintexts corresponding to *t* ciphertexts *δ*_*i*_ are equal. By calculating $f_{j,k}^{i}$, we can get the non-homogeneous linear equation set
    $$\begin{array}{c} \left\{ \begin{array}{c} f_{k}^{1}\left(X_{1}\right)=f_{0,k}^{1}+f_{1,k}^{1}X_{1}+f_{2,k}^{1}{X_{1}}^{2}+\cdots +f_{k-1,k}^{1}X_{1}^{k-1}\\ f_{k}^{2}\left(X_{2}\right)=f_{0,k}^{2}+f_{1,k}^{2}X_{2}+f_{2,k}^{2}{X_{2}}^{2}+\cdots +f_{k-1,k}^{2}X_{2}^{k-1}\\ \;\;\;\vdots \\ f_{k}^{k}\left(X_{k}\right)=f_{0,k}^{k}+f_{1,k}^{k}X_{1}+f_{2,k}^{k}{X_{k}}^{2}+\cdots +f_{k-1,k}^{k}X_{k}^{k-1} \end{array}.\right. \end{array}$$
    Let $f_{j,k}^{i_{1}}=f_{j,k}^{i_{2}}$ where *i*_1_, *i*_2_ ∈ {1, 2, ⋯, *k*} and *j* ∈ {1, 2, ⋯, *k* − 1}. If $f_{0,k}^{i},$, $f_{k-1,k}^{i}$ is regarded as the solution of the equation set. And *X*_*i*_ is regarded as the coefficient. det(*V*) ≠ 0 can be known according to Kramer’s rule and Vandermonde determinant. The unique solution $f_{0,k}^{i},f_{1,k}^{i},\cdots ,f_{k-1,k}^{i}$ of the equation set can be obtained.
  - For each ciphertext *δ*_*i*_ = (*C*_*i*__,1_, *C*_*i*,__2_, *C*_*i*_, _3_, *C*_*i*,__4_, *k*_*i*_), verify whether the equation $C_{i,4}=H_{4}(C_{1}\left|\right|C_{2}\left|\right|C_{3}\left|\right|f_{0,k}^{i}\left|\right|f_{1,k}^{i}\left||\cdots |\right|f_{k-1,k}^{i}||td_{i}C_{i,1}\left||k\right)$ holds.
    If the equation is true for every *δ*_*i*_, it represents *m*_1_ = *m*_2_ = ⋯ = *m*_*t*_, and the test result 1 is returned. Otherwise, 0 is returned.

## Correctness analysis

**Theorem 1.**The unsigncryption algorithm is correct.

**Proof.** The correctness of the unsigncryption algorithm can be verified by the following two equations.

1. After receiving the ciphertext *δ* = (*C*_1_, *C*_2_, *C*_3_, *C*_4_, *k*), the data user can get *m*′ by calculating *m*′ ∥*r*′ = *H*_2_ (*Y*′) ⊕ *H*_2_ (*R*′) ⊕ *C*_2_. Eq (4) holds.
  $$\begin{array}{c} \begin{array}{c} m^{{}^{\prime }}\left|\right|r^{{}^{\prime }}=H_{2}\left(Y{}^{\prime }\right)⊕H_{2}\left(R{}^{\prime }\right)⊕C_{2}=H_{2}\left(SK_{c1}PK_{p}\right)⊕H_{2}\left(SK_{c2}C_{1}\right)⊕C_{2}\\ \;\;\;\;\;\;\;=m||r \end{array} \end{array}$$ (4)
2. The data user can calculate $X^{{}^{\prime }}\left|\right|{f^{{}^{\prime }}}_{k}\left(X\right)||\cdots ||{f^{{}^{\prime }}}_{n}\left(X\right)=C_{3}⊕H_{3}\left(SK_{c2}C_{1}\right)$ to verify the legitimacy of the message and signature. Eq (5) holds.
  $$\begin{array}{c} \begin{array}{c} X{}^{\prime }\left|\right|{f^{{}^{\prime }}}_{k}\left(X\right)||\cdots ||{f^{{}^{\prime }}}_{n}\left(X\right)=C_{3}⊕H_{3}\left(R{}^{\prime }\right)\\ =\left(X|\right|f_{k}\left(X\right)||\cdots ||f_{n}\left(X\right))⊕H_{3}\left(rPK_{c2}\right)⊕H_{3}\left(SK_{c2}C_{1}\right)\\ =\left(X|\right|f_{k}\left(X\right)||\cdots ||f_{n}\left(X\right))⊕H_{3}\left(rSK_{c2}P\right)⊕H_{3}\left(SK_{c2}rP\right)\\ =X||f_{k}\left(X\right)||\cdots ||f_{n}\left(X\right) \end{array} \end{array}$$ (5)

Through the above verification, theorem 1 is established.

**Theorem 2.**The Test algorithm is correct.

**Proof.** The correctness of the Test algorithm can be verified by the following equations.

Given t ciphertexts *δ*_*i*_ = (*C*_*i*__,1_, *C*_*i*,2_, *C*_*i*_, _3_, *C*_*i*,4_, *k*_*i*_). Let *k* = max{*k*_1_, *k*_2_, ⋯, *k*_*t*_}. If *k* ≤ min{*t*, *n*}, calculate $X_{i}\left|\right|f_{i,k_{i}}\left(X_{i}\right)\left||\cdots |\right|f_{i,n}\left(X_{i}\right)=C_{i,3}⊕H_{3}\left(td_{i}C_{i,1}\right)$.

Assume that the plaintexts of *t* ciphertexts *δ*_*i*_ are *m*_1_, *m*_2_, ⋯, *m*_*t*_ respectively.

1. When the plaintexts corresponding to the tested *t* ciphertexts are equal, the correctness of the Test algorithm is proved as follows.
  If *m*_1_ = *m*_2_ = ⋯ = *m*_*t*_, we must have $f_{j,k}^{i_{1}}=f_{j,k}^{i_{2}}$ where *i*_1_, *i*_2_ ∈ {1, 2, ⋯, *t*} and *j* ∈ {0, 2, ⋯, *t* − 1}. Let $f_{j,k}^{i}=f_{j,k}^{1}$. We can get the equation set Eq (6).
  $$\begin{array}{c} \left\{ \begin{array}{c} f_{k}^{1}\left(X_{1}\right)=f_{0,k}^{1}+f_{1,k}^{1}X_{1}+\cdots +f_{k-1,k}^{1}X_{1}^{k-1}\\ f_{k}^{2}\left(X_{2}\right)=f_{0,k}^{1}+f_{1,k}^{1}X_{2}+\cdots +f_{k-1,k}^{1}X_{2}^{k-1}\\ \;\;\;\vdots \\ f_{k}^{k}\left(X_{k}\right)=f_{0,k}^{1}+f_{1,k}^{1}X_{k}+\cdots +f_{k-1,k}^{1}X_{k}^{k-1} \end{array}\right. \end{array}$$ (6)
  If $f_{0,k}^{1},f_{1,k}^{1},\cdots f_{k-1,k}^{1}$ is regarded as the solution of the equation set, and *X*_*i*_ is regarded as a coefficient. The equation set corresponds to the Vandermonde matrix Eq (7).
  $$\begin{array}{c} V=[\begin{array}{ccccc} 1 & X_{1} & X_{1}^{2} & \cdots & X_{1}^{k-1}\\ 1 & X_{2} & X_{2}^{2} & \cdots & X_{2}^{k-1}\\ \vdots & \vdots & \vdots & \vdots & \vdots \\ 1 & X_{k} & X_{k}^{2} & \cdots & X_{k}^{k-1} \end{array}] \end{array}$$ (7)
  The determinant of *V* is $\text{det}\left(V\right)=\prod_{1\leq i\leq j\leq k}(X_{i}-X_{j})$. Due to the randomness of *X*_*i*_, the probability of det(*V*) = 0 is $\frac{1}{q(q-1)\cdots (q-k+1)}.$ The equation set has a unique solution $f_{0,k}^{1},f_{1,k}^{1},\cdots f_{k-1,k}^{1}$ when det(*V*) ≠ 0 from Cramer’s rule. For each ciphertext *δ*_*i*_, the equation $C_{i,4}=H_{4}(C_{1}\left|\right|C_{2}\left|\right|C_{3}\left|\right|f_{0,k}^{1}\left|\right|f_{1,k}^{1}\left||\cdots |\right|f_{k-1,k}^{1}||td_{i}C_{i,1}\left||k\right)$ holds. We can get *Test*(*para*, *δ*_1_, *δ*_2_, ⋯, *δ*_*k*_, *td*_1_, *td*_2_, ⋯, *td*_*k*_) = 1.
2. When the plaintexts corresponding to the tested *t* ciphertexts are not equal, the correctness of the Test algorithm is proved as follows.
  If *m*_1_ ≠ *m*_2_ = ⋯ = *m*_*t*_, there is $f_{j,k}^{1}\neq f_{j,k}^{i_{1}}=f_{j,k}^{i_{2}}$, where *i*_1_, *i*_2_ ∈ {2, 3, ⋯, *t*} and *j* ∈ {0, 2, ⋯, *t* − 1}. We can obtain the equation set Eq (8).
  $$\begin{array}{c} \left\{ \begin{array}{c} f_{k}^{1}\left(X_{1}\right)=f_{0,k}^{1}+f_{1,k}^{1}X_{1}+\cdots +f_{k-1,k}^{1}X_{1}^{k-1}\\ f_{k}^{2}\left(X_{2}\right)=f_{0,k}^{2}+f_{1,k}^{2}X_{2}+\cdots +f_{k-1,k}^{2}X_{2}^{k-1}\\ \;\;\;\vdots \\ f_{k}^{k}\left(X_{k}\right)=f_{0,k}^{2}+f_{1,k}^{2}X_{k}+\cdots +f_{k-1,k}^{2}X_{k}^{k-1} \end{array}\right. \end{array}$$ (8)
  Let $f_{j,k}^{2}=f_{j,k}^{1}=f{}_{j,k}^{1}*$ where *j* ∈ {0, 2, ⋯, *t* − 1}. The unique solution $f{{}_{0,k}^{1}}^{*},f{{}_{1,k}^{1}}^{*},\cdots ,f{{}_{k-1,k}^{1}}^{*}$ can be obtained. It cannot make the Eqs (9) and (10) hold at the same time.
  $$\begin{array}{c} \begin{array}{c} C_{1,4}=H_{4}(C_{1,1}\left|\right|C_{1,2}\left|\right|C_{1,3}\left|\right|f_{0,k_{1}}^{1}||f{}_{1,k_{1}}^{1}*||\cdots ||f{}_{k_{1}-1,k_{1}}^{1}*||td_{1}C_{1,1}\left|\right|k_{1}) \end{array} \end{array}$$ (9)
  $$\begin{array}{c} \begin{array}{c} C_{2,4}=H_{4}(C_{2,1}\left|\right|C_{2,2}\left|\right|C_{2,3}\left|\right|f_{0,k_{2}}^{2}||f{}_{1,k_{2}}^{1}*||\cdots ||f{}_{k_{2}-1,k_{2}}^{1}*||td_{2}C_{2,1}\left|\right|k_{2}) \end{array} \end{array}$$ (10)

Through the above verification, theorem 2 is established.

## Security proofs

### Confidentiality

**Theorem 3.** If an adversary $A_{1}$ can win the Game 1 in PPT with a non-negligible advantage *ε*_1_ after $q_{h_{i}}\left(i=1,2,3,4\right)$ *H*_*i*_ queries, *q*_*d*_ partial private key queries, *q*_sc_ signcryption queries and *q*_usc_ unsigncryption queries, the challenger C can solve the CDH problem with the nonnegligible advantage $\varepsilon_{1}^{{}^{\prime }}$ as show in Eq (11).

$$\begin{array}{c} {\varepsilon_{1}}^{{}^{\prime }}=\left(1-\frac{q_{d}}{q_{h_{1}}}\right)\left(1-\frac{q_{sc}\left(q_{h_{2}}+q_{h_{3}}+q_{h_{4}}\right)}{2^{\lambda }}\right)\left(1-\frac{q_{usc}}{2^{\lambda }}\right)\varepsilon_{1} \end{array}$$ (11)

**Proof:** C is a challenger to solve the CDH problem. $A_{1}$ is a Type-1 adversary. Given a challenge example (*P*, *aP*, *bP*) where $a,b\in Z_{q}^{*}$. C and $A_{1}$ interact as follows.

**Setup:** C executes the setup algorithm to output the system parameter *para* = {λ, *G*, *q*, *P*, *PK*, *H*_1_, *H*_2_, *H*_3_, *H*_4_, *n*}.

**Phase 1:** C needs to maintain initially empty lists *L*_*hi*_, *i* = 1, 2, 3, 4, *L*_*d*_, *L*_*sk*_, *L*_*pk*_ and *L*_*td*_ to record the query results of $A_{1}$.

- ***H*_1_ query:** When receiving the query with *ID*_*i*_ submitted by $A_{1}$, C searches for whether there is (*ID*_*i*_, *h*_1_) in *L*_*h*1_. When it exists, C returns *h*_1_ to $A_{1}$. Otherwise, C slelects $h_{1}\in Z_{q}^{*}$ randomly and returns to $A_{1}$. C inserts (*ID*_*i*_, *h*_*i*_) into *L*_*h*1_ finally.
- ***H*_2_ query:** When receiving the query with *R*_*i*_ submitted by $A_{1}$, C searches for whether there is (*R*_*i*_, *h*_2_) in *L*_*h*2_. When it exists, C returns *h*_2_ to $A_{1}$. Otherwise, C randomly selects *h*_2_ = {0, 1}^2*l*^ and returns to $A_{1}$. And C inserts (*R*_*i*_, *h*_2_) into *L*_*h*2_.
- ***H*_3_ query:** When receiving the query with (*r*_*i*_, *PK*_*ic*2_) submitted by $A_{1}$, C searches for whether there is (*r*_*i*_, *PK*_*ic*2_, *h*_3_) in *L*_*h*3_. When it exists, C returns *h*_3_ to $A_{1}$. Otherwise, C randomly selects *h*_3_ = {0, 1}^*nl*^ and returns to $A_{I}$. And C inserts (*r*_*i*_, *PK*_*ic*2_, *h*_3_) into *L*_*h*3_.
- ***H*_4_ query:** When receiving the query with (*C*_1,*i*_, *C*_2,*i*_, *C*_3,*i*_, *f*_*i*,*k*_, *r*_*i*_, *PK*_*ic*2_, *k*_*i*_) submitted by $A_{1}$, C searches for whether there is the corresponding *h*_4_ in *L*_*h*4_. When it exists, C returns *h*_4_ to $A_{1}$. Otherwise, C selects *h*_4_ = {0, 1}^*l*^ randomly and returns to $A_{1}$. Then C inserts (*C*_1,*i*_, *C*_2,*i*_, *C*_3,*i*_, *f*_*i*,*k*_, *r*_*i*_, *PK*_*ic*2_, *k*_*i*_, *h*_4_) into *L*_*h*4_.
- **Partial private key query:** When receiving the query with *ID*_*ci*_ from $A_{1}$, if (*ID*_*ci*_, *SK*_*ic*1_) exists, C returns it to $A_{1}$. Otherwise, C executes CLC-PGen algorithm to generate *SK*_*ic*1_ and return to $A_{1}$. C inserts (*ID*_*ci*_, *SK*_*ic*1_) into *L*_*d*_.
- **Private key query:** When receiving the query with *ID*_*ci*_ from $A_{1}$, if (*ID*_*ci*_, *SK*_*ci*_) exists, C returns it to $A_{1}$. Otherwise, C executes CLC-CGen algorithm to generate *SK*_*ci*_ and return to $A_{1}$. C inserts (*ID*_*ci*_, *SK*_*ci*_) into *L*_*sk*_.
- **Public key query:** When receiving the query with *ID*_*ci*_ from $A_{1}$, if (*ID*_*ci*_, *PK*_*ci*_) exists, C returns it to $A_{1}$. Otherwise, C executes CLC-PGen algorithm to generate *PK*_*ci*_ and return to $A_{1}$. C inserts (*ID*_*ci*_, *PK*_*ci*_) into *L*_*pk*_.
- **Replace public key query:** $A_{1}$ can select any public key $P{K_{c2}}^{*}$ to replace the user’s original public key *PK*_*c*2_.
- **Trapdoor query:** When receiving the query with *ID*_*ci*_ from $A_{1}$, if (*ID*_*ci*_, *td*_*ci*_) exists, C returns it to $A_{1}$. Otherwise, C executes Trapdoor algorithm to generate *td*_*ci*_ and return to $A_{1}$, and inserts (*ID*_*ci*_, *td*_*ci*_) into *L*_*td*_.
- **Signcryption query:** When receiving the query with (*m*_*i*_, *ID*_*pi*_, *ID*_*ci*_) submitted by $A_{1}$, C executes the Signcryption algorithm to obtain the ciphertext *δ*_*i*_, and returns it to $A_{1}$.
- **Unsigncryption query:** When receiving the query with (*ID*_*pi*_, *ID*_*ci*_, *δ*_*i*_) submitted by $A_{1}$, C executes the Unsigncryption algorithm to obtain the plaintext *m*_*i*_, and returns it to $A_{1}$.

**Challenge:** $A_{1}$ submits the sender’s identity $I{D_{p}}^{*}$, receiver’s identity $I{D_{c}}^{*}$, and two plaintexts *m*_0_ and *m*_1_ of the same length to C. $A_{1}$ has never asked for the private key for $I{D_{c}}^{*}$. C randomly selects $a\in Z_{q}^{*}$ as the secret value of $I{D_{c}}^{*}$ and calculates $PK_{c2}^{*}=aP$. Then C randomly selects *ξ* ∈ {0, 1} and performs the following calculations.

- Calculate *f*_0,*n*_ = *H*_1_(*m*_*ξ*_||*n*) and *f*_*i*,*n*_ = *H*_1_(*m*_*ξ*_||*n*||*f*_0,*n*_||⋯||*f*_*i* − 1, *n*_) where *i* ∈ {1, 2, ⋯, *n* − 1}.
- Calculate *f*_*i*,*j*_ = *H*_1_(*f*_*i*,*j*+1_) where *i* ∈ {*k*, *k* + 1, ⋯, *n* − 1} and j ∈ {0, 1, ⋯, *i* − 1}.
- Calculate *f*_*i*_(*x*) = *f*_0,*i*_ + *f*_1,*i*_ *x* + ⋯ + *f*_*i* − 1, *i*_ *x*^*i*−1^ where *i* ∈ {*k*, *k* + 1, ⋯, *n*}.
- Randomly select $b,X\in Z_{q}^{*}$, and calculate $Y^{*}=SK_{p}^{*}(PKH_{1}\left(ID_{c}^{*}\right)+PK_{c1}^{*}$ and $R^{*}=bPK_{c2}^{*}$.
- Calculate $C_{1}^{*}=bP$, $C_{2}^{*}=(m_{\xi }\left||b\right)⊕H_{2}\left(Y^{*}\right)⊕H_{2}\left(R^{*}\right)$, $C_{3}^{*}=\left(X|\right|f_{k}\left(X\right)||\cdots ||$ *f*_*n*_(*X*))⊕*H*_3_(R*) and $C_{4}^{*}=H_{4}(C_{1}^{*}\left|\right|C_{2}^{*}\left|\right|C_{3}^{*}\left|\right|f_{0,k}\left|\right|f_{1,k}\left||\cdots |\right|f_{k-1,k}\left|\right|R^{*}\left||k\right)$.
-
C returns $\delta^{*}=\left(C_{1}^{*},C_{2}^{*},C_{3}^{*},C_{4}^{*},k\right)$ to $A_{1}$.

**Phase 2:** $A_{1}$ continues to perform the queries after receiving δ*, but $A_{1}$ cannot query the private key of the *ID*_*ci*_, nor can it perform unsigncryption query on δ*.

**Guess:** $A_{1}$ outputs the guess value ξ*. If ξ* = *ξ*, $A_{1}$ wins the game. C will select (*R*_*i*_, *H*_2_(*R*_*i*_)) from the list *L*_*h*2_ and take *R*_*i*_ = *abP* as the solution of the CDH problem. However, there is currently no effective way to solve the CDH problem. Theorem 3 is proved.

**Theorem 4.** If an adversary $A_{2}$ can win the Game 2 in PPT with a non-negligible advantage *ε*_2_ after $q_{h_{i}}\left(i=1,2,3,4\right)$ *H*_*i*_ queries, *q*_*d*_ partial private key queries, *q*_sc_ signcryption queries and *q*_usc_ unsigncryption queries, the challenger C can solve the CDH problem with the advantage $\varepsilon_{2}^{{}^{\prime }}$ as show in Eq (12).

$$\begin{array}{c} {\varepsilon_{2}}^{{}^{\prime }}=\left(1-\frac{q_{d}}{q_{h_{1}}}\right)\left(1-\frac{q_{sc}\left(q_{h_{2}}+q_{h_{3}}+q_{h_{4}}\right)}{2^{\lambda }}\right)\left(1-\frac{q_{usc}}{2^{\lambda }}\right)\varepsilon_{2} \end{array}$$ (12)

The proof process is similar to Theorem 3 and will not be repeated here.

### Unforgeability

**Theorem 5.** If an adversary F can win the Game 3 in PPT with a non-negligible advantage *ε*_3_ after $q_{h_{i}}\left(i=1,2,3,4\right)$ *H*_*i*_ queries, *q*_*pk*_ public key queries and *q*_sc_ signcryption queries, the challenger C can solve the CDH problem with the advantage $\varepsilon_{3}^{{}^{\prime }}$ as show in Eq (13).

$$\begin{array}{c} {\varepsilon_{3}}^{{}^{\prime }}=\left(1-\frac{q_{pk}}{2^{\lambda }}\right)\left(1-\frac{q_{sc}\left(q_{h_{2}}+q_{h_{3}}+q_{h_{4}}\right)}{2^{\lambda }}\right)\varepsilon_{3} \end{array}$$ (13)

**Proof:** C is a challenger to solve the difficult problems of CDH. F is an adversary. C selects $ID_{p}^{*}$ as the challenge identity. Given a challenge example (*P*, *aP*, *bP*) where $a,b\in Z_{q}^{*}$. C and F interact as follows.

**Setup:** C randomly selects $a\in Z_{q}^{*}$ and calculates *PK* = *aP*. C outputs the system parameter *para* = {λ, *G*, *q*, *P*, *PK*, *H*_1_, *H*_2_, *H*_3_, *H*_4_, *n*}.

**Training:** The same queries as Theorem 3 will not be repeated here. The different queries are described below.

- **Key query:** When receiving the query with *ID*_*pi*_ submitted by F, C executes the PKI-Gen algorithm to generate (*SK*_*p*_, *PK*_*p*_) and return to F if $ID_{pi}\neq ID_{p}^{*}$. Otherwise, C randomly selects $b\in Z_{q}^{*}$ and calculates *PK*_*p*_ = *bP*. Then C renturns *PK*_*p*_ to F.
- **Signcryption query:** When receiving the query with (*ID*_*pi*_, *ID*_*ci*_, *m*_*i*_) submitted by F, C executes the signcryption algorithm to generate $\delta_{i}^{*}$ and return to F if $ID_{pi}\neq ID_{p}^{*}$. Otherwise, C performs the following operations.
  - Calculate *f*_0,*n*_ = *H*_1_(*m*_*i*_||*n*) and *f*_*i*,*n*_ = *H*_1_(*m*_*i*_||*n*||*f*_0,*n*_||⋯||*f*_*i* − 1, *n*_) where *i* ∈ {1, 2, ⋯, *n* − 1}.
  - Calculate *f*_*i*,*j*_ = *H*_1_(*f*_*i*,*j*+1_) where *i* ∈ {*k*, ⋯, *n* − 1} and j ∈ {0, ⋯, *i* − 1}.
  - Calculate *f*_*i*_(*x*) = *f*_0,*i*_ + *f*_1,*i*_ *x* + ⋯ + *f*_*i* − 1, *i*_ *x*^*i*−1^ where *i* ∈ {*k*, *k* + 1, ⋯, *n*}.
  - Randomly select $r,X^{\prime }\in Z_{q}^{*}$. Calculate Y* = *b*(*PKH*_1_(*ID*_*ci*_) + *PK*_*ic*1_) and R* = *rPK*_*ic*2_.
  - Calculate $C_{1}^{*}=rP$, $C_{2}^{*}=(m_{i}\left||r\right)⊕H_{2}\left(Y^{*}\right)⊕H_{2}\left(R^{*}\right)$, $C_{3}^{*}=\left(X|\right|f_{k}\left(X\right)\left|\right|\cdots$ ||*f*_*n*_(*X*))⊕*H*_3_(R*) and $C_{4}^{*}=H_{4}(C_{1}^{*}\left|\right|C_{2}^{*}\left|\right|C_{3}^{*}\left|\right|f_{0,k}\left|\right|f_{1,k}\left||\cdots |\right|f_{k-1,k}\left|\right|R^{*}\left|\right|k_{i})$.
  - Return $\delta^{*}=\left(C_{1}^{*},C_{2}^{*},C_{3}^{*},C_{4}^{*},k_{i}\right)$ to F.

**Forgery:** F outputs a forged ciphertext $\delta^{{}^{\prime }}=\left(C_{1}^{{}^{\prime }},C_{2}^{{}^{\prime }},C_{3}^{{}^{\prime }},C_{4}^{{}^{\prime }},k_{i}\right)$ for *m*_*i*_. If the forgery is successful, C can select $\left(Y^{\prime },H_{2}\left(Y^{\prime }\right)\right)$ from the list *L*_*h*2_ and take $abP=\frac{Y^{\prime }-R^{\prime }}{H_{1}\left(ID_{ci}\right)}$ as the solution of the CDH problem. However, there is currently no effective way to solve the problem. Theorem 5 is proved.

### Number security

In this section, we proved the number security of our scheme based on the definition of number security in reference [28].

**Theorem 6.** If there is an adversary A, after $q_{h_{i}}\left(i=1,2,3,4\right)$ *H*_*i*_ queries, *q*_*td*_ trapdoor queries, *q*_sc_ signcryption queries and *q*_usc_ unsigncryption queries, can determine whether the underlying plaintext corresponding to *t* < *K* ciphertext *δ*_*i*_ = (*C*_*i*,1_, *C*_*i*,2_, *C*_*i*,3_, *C*_*i*,4_, *k*_*i*_) is equal in PPT with a non-negligible advantage *ε*_4_, where *k* = max{*k*_1_, *k*_2_, ⋯, *k*_*t*_}. C can solve the problem of CDH with the advantage ${\varepsilon_{4}}^{\prime }$ as show in Eq (14).

$$\begin{array}{c} {\varepsilon_{4}}^{{}^{\prime }}=\left(1-\frac{q_{td}}{2^{\lambda }}\right)\left(1-\frac{q_{sc}\left(q_{h_{2}}+q_{h_{3}}+q_{h_{4}}\right)}{2^{\lambda }}\right)\left(1-\frac{q_{usc}}{2^{\lambda }}\right)\varepsilon_{4} \end{array}$$ (14)

**Proof:** There are the following two ways to determine for A.

1.
A can determine by obtaining and comparing the plaintexts *m*_1_, *m*_2_, ⋯, *m*_*t*_.
  In subsection Confidentiality, we have proved the confidentiality of our scheme. So this way is not feasible for A.
2.
A can determine by obtaining the value of ${f_{i,k^{{}^{\prime }}}}^{l}$, where *i* ∈ {1, 2, ⋯, *t* − 1}, *l* ∈ {1, 2, ⋯, *t*} and $k_{i}<k^{{}^{\prime }}<n$. For this way, we do the following analysis.

For *t* ciphertexts *δ*_1_, *δ*_2_, ⋯, *δ*_*t*_, A is allowed to perform public key queries and trapdoor queries. So it can calculate $X_{i}\left|\right|f_{k_{i}}\left(X_{i}\right)\left||\cdots |\right|f_{n}\left(X_{i}\right)=C_{i,3}⊕H_{3}\left(td_{ci}C_{i,1}\right)$ and $f_{j}^{i}\left(X_{i}\right)=f_{0,j}^{i}+f_{1,j}^{i}X_{i}+\cdots +f_{j-1,j}^{i}{X_{i}}^{j-1}$ where *k*_*i*_ < *j* < *n*. Let *k* = max{*k*_1_, ⋯, *k*_*t*_}. A can get the equation set Eq (15).

$$\begin{array}{c} \left\{ \begin{array}{c} f_{k}^{1}\left(X_{1}\right)=f_{0,k}^{1}+f_{1,k}^{1}X_{1}+f_{2,k}^{1}{X_{1}}^{2}+\cdots +f_{k-1,k}^{1}X_{1}^{k-1}\\ f_{k}^{2}\left(X_{2}\right)=f_{0,k}^{2}+f_{1,k}^{2}X_{2}+f_{2,k}^{2}{X_{2}}^{2}+\cdots +f_{k-1,k}^{2}X_{2}^{k-1}\\ \;\;\;\vdots \\ f_{k}^{t}\left(X_{t}\right)=f_{0,k}^{t}+f_{1,k}^{t}X_{t}+f_{2,k}^{t}{X_{t}}^{2}+\cdots +f_{k-1,k}^{t}X_{t}^{k-1} \end{array}\right. \end{array}$$ (15)

Since *X*_1_, *X*_2_, ⋯, *X*_*t*_ are randomly selected by users, the probability of non-linear correlation of *t* equations is $p=\frac{1}{q(q-1)\cdots (q-k+1)}$. Let $f_{j,k}^{i}=f_{j,k}^{1}$, where *i* ∈ {1, 2, ⋯, *t*} and *j* ∈ {0, 1, ⋯, *k* − 1}. If $f_{0,k}^{1},f_{1,k}^{1},\cdots f_{k-1,k}^{1}$ is regarded as an independent variable, and *X*_*i*_ is regarded as a coefficient of the equations, the equation set consisting of *t* equations with *k* independent variables can be obtained. Because of *k* > *t*, there is no solution to make the equation set true. So this way is not feasible for A.

In summary, the HSC-MET scheme satisfies the number security. Theorem 6 is proved.

## Scheme analysis

### Functional analysis

Table 3 summarizes the functional properties, confidentiality, and unforgeability analyses of our scheme. can be seen in Tables 1 and 3. To begin, in comparison to the references [13–20], our scheme incorporates the MET function to achieve safe and efficient cloud data retrieval, which is more appropriate for application scenarios involving large amounts of data. Second, when compared to the schemes in [22–27], our scheme overcomes the limitation of only supporting pairwise grouping for ciphertext equality tests, making it more appropriate for multi-user and multi-ciphertext application scenarios. Third, unlike [13, 15, 17, 18, 20, 22–27], our scheme does not use bilinear pairing and has lower computational costs. Furthermore, our scheme has higher confidentiality than [13, 22–24, 27, 28]. Finally, unlike [22–24, 28], our scheme has unforgeability and introduces heterogeneous signcryption technology to ensure the confidentiality, and integrity of data, and realizes the secure communication between heterogeneous cryptosystems.

**Table 3:** Characteristics of our scheme.

Scheme	ET	MET	SC	HSC	Without bilinear pairing	Cryptosystem	Confidentiality	Unforgeability
Our scheme	✓	✓	✓	✓	✓	PKI→CLC	IND-CCA2	EUF-CMA

×: not supported;

✓: supported.

### Performance analysis

**Fig 2:**
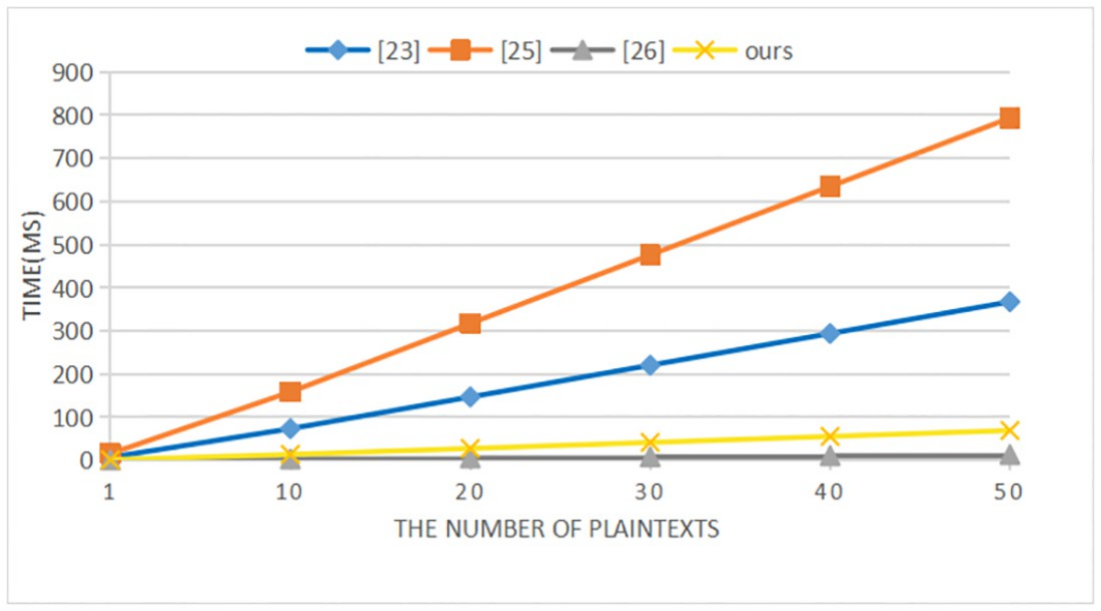
Computational cost of signcryption/encryption.

**Fig 3:**
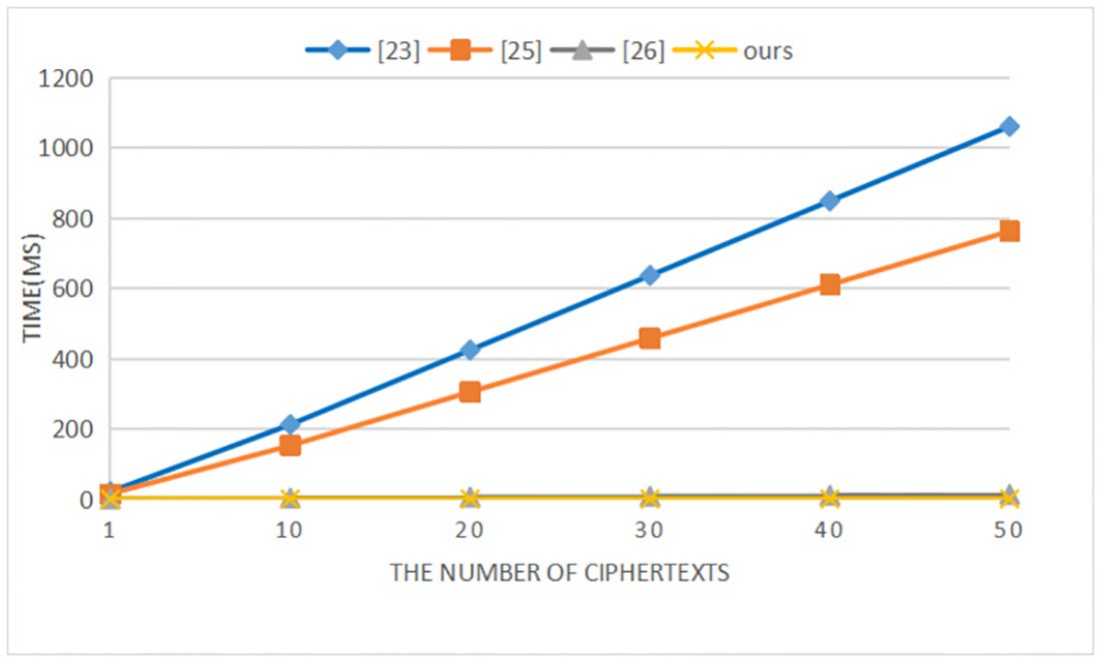
Computational cost of unsigncryption/decryption.

**Fig 4:**
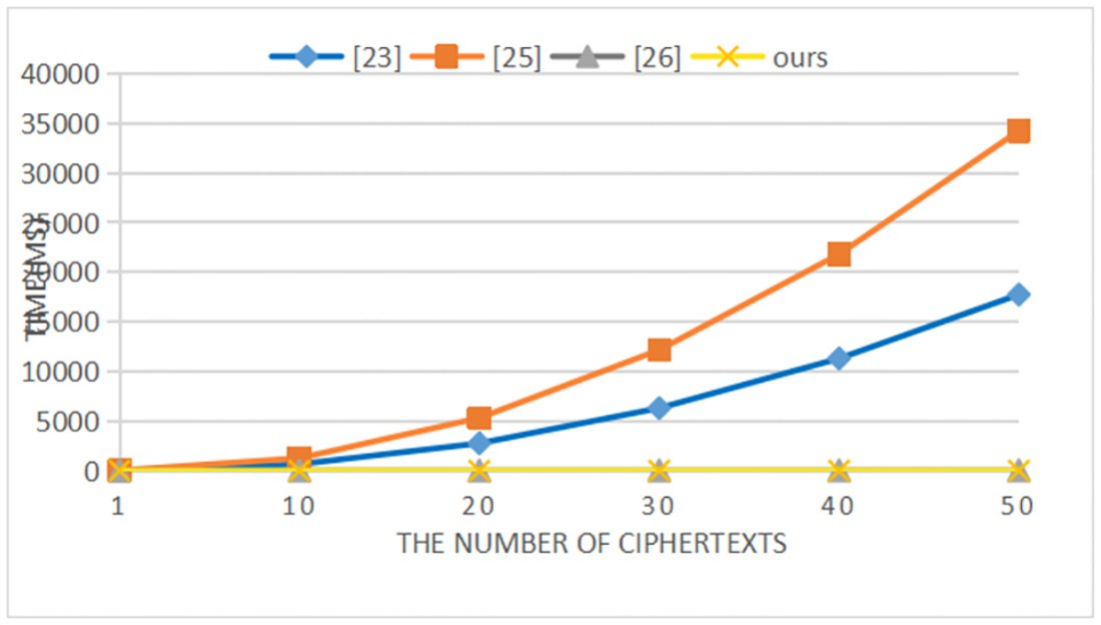
Computational cost of test.

Our scheme is compared with the schemes in references [25, 27, 28] in terms of performance. Reference [28] uses the traditional public key encryption scheme. We use a PC equipped with Intel Core i7–7500u CPU@3.5GHz, 8G memory, and Windows 10 for simulation. The representative symbols and their meaning and computational time are shown in Table 4. The computational cost of each comparison scheme is shown in Table 5. With the increase of plaintexts/ciphertexts, the computational costs of our scheme and the comparison schemes in the signcryption/encryption, unsigncryption/decryption, and test phases are shown in Figs 2–4 respectively.

**Table 4:** Notations.

Symbols	Representations	Time(ms)
*k*	The number of ciphertexts that can run the Test algorithm	−−
*T*_*h*_	The time it takes to run a hash operation	0.0014
*T*_*a*_	The time it takes to run a point-add operation in group *G*_1_	1.3667
*T*_*m*_	The time it takes to run a multiplicative operation in group *G*_1_	0.0032
*T*_*e*_	The time it takes to run an exponential operation in group *G*_2_	0.2549
*T*_*p*_	The time it takes to run a bilinear pairing operation	6.9841

**Table 5:** Comparison of computational cost.

Schemes	Signcryption/Encryption	Unsigncryption/Decryption	Test
[25]	5*T*_*h*_ + 5*T*_*a*_ + 2*T*_*e*_ = 7.3503*ms*	4*T*_*h*_ + *T*_*e*_ + 3*T*_*p*_ = 21.2122*ms*	2*T*_*h*_ + 2*T*_*e*_ + 2*T*_*p*_ = 14.4808*ms*
[27]	4*T*_*h*_ + *T*_*a*_ + 2*T*_*e*_ + 5*T*_*m*_ + 3*T*_*p*_ = 15.8663*ms*	4*T*_*h*_ + 5*T*_*e*_ + 2*T*_*p*_ = 15.2483*ms*	2*T*_*h*_ + 2*T*_*m*_ + 4*T*_*p*_ = 27.9456*ms*
[28]	4*T*_*h*_ + 3*T*_*e*_ = 0.0073*ms*	3*T*_*h*_ + 2*T*_*e*_ = 0.514*ms*	2*T*_*h*_ + 2*T*_*e*_ = 0.5216*ms*
ours(k = 1)	5*T*_*h*_ + *T*_*a*_ + 5*T*_*m*_ = 1.3896*ms*	5*T*_*h*_ + 3*T*_*m*_ = 0.0166*ms*	2*T*_*h*_ + 4*T*_*m*_ = 0.0156*ms*

In the signcryption/encryption phase, it can be seen from Table 5 and Fig 2 that compared with the schemes in [25] and [27], our scheme does not have bilinear pairing operations, which greatly reduces the computational cost. Although compared with the scheme in [28], the computational cost of our scheme is higher, our scheme not only achieves confidentiality, but also satisfies non-repudiation. And our scheme supports communication between heterogeneous cryptosystem. In the unsigcryption/decryption and test phases, Figs 3 and 4 clearly show that our scheme has lower computational costs than the schemes in [25, 27, 28]. When the number of ciphertexts reaches 20, the computational efficiency in unsigcryption/decryption phase of our scheme is approximately 2000 times, 1500 times and 30 times that of the three comparison schemes. And as the number of ciphertexts increases, the advantages of our scheme become more obvious in the test phase.

## Conclusion

We proposed the HSC-MET scheme to overcome the problems in the existing schemes, such as not supporting the communication between heterogeneous cryptosystems, high computational overhead, and low efficiency of ciphertext retrieval. Our scheme uses HSC technology to realize secure communication from PKI to CLC. The scheme has no bilinear pairing operation, which greatly reduces the computational cost and improves communication efficiency. In addition, the multi-ciphertext equality test technology is introduced to realize the simultaneous retrieval of multiple ciphertexts by multiple users, which reduces the computational cost of the ciphertext equality test in the multi-user scenario. Under the ROM, we proved the confidentiality, unforgeability, and number security of the HSC-MET scheme based on the CDH problem. Finally, we compared the scheme with several similar schemes. The results show that our scheme not only has more functional features and higher security but also has lower computational costs in signcryption, unsigncryption, and test phases. However, our scheme’s security is proved under the random oracle model, which is not universal in reality more or less. In the future, we will further investigate the security under the standard model to make the HSC-MET scheme more practical.

## Supporting information

S1 Fig Cloud server. Image URL: https://www.iconfont.cn/search/index?searchType=iconq=cloud.
(TIF)

S2 Fig KGC. Image URL: https://www.iconfont.cn/search/index?searchType=iconq=sever.
(TIF)

S3 Fig UAVs. Image URL: https://www.iconfont.cn/search/index?searchType=iconq=UAV.
(TIF)

S4 Fig Data users. Image URL: https://www.iconfont.cn/search/index?searchType=iconq=user.
(TIF)

S5 Fig CA. Image URL: https://www.iconfont.cn/search/index?searchType=iconq=host.
(TIF)
