# Supplementary figures and images for: HSC-MET: Heterogeneous signcryption scheme supporting multi-ciphertext equality test for Internet of Drones
Source: PLoS One. 2022 Sep 29;17(9):e0274695. doi: 10.1371/journal.pone.0274695 (PMC9522033; doi:10.1371/journal.pone.0274695)

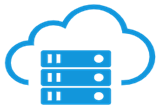

Supplement: S1 Fig — Image URL: https://www.iconfont.cn/search/index?searchType=iconq=cloud. (TIF) [file pone.0274695.s001.tif]

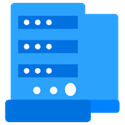

Supplement: S2 Fig — Image URL: https://www.iconfont.cn/search/index?searchType=iconq=sever. (TIF) [file pone.0274695.s002.tif]

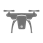

Supplement: S3 Fig — Image URL: https://www.iconfont.cn/search/index?searchType=iconq=UAV. (TIF) [file pone.0274695.s003.tif]

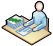

Supplement: S4 Fig — Image URL: https://www.iconfont.cn/search/index?searchType=iconq=user. (TIF) [file pone.0274695.s004.tif]

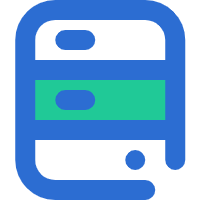

Supplement: S5 Fig — Image URL: https://www.iconfont.cn/search/index?searchType=iconq=host. (TIF) [file pone.0274695.s005.tif]
